# Conspicuity of Malignant Lesions on PET/CT and Simultaneous Time-Of-Flight PET/MRI

**DOI:** 10.1371/journal.pone.0167262

**Published:** 2017-01-19

**Authors:** Ryogo Minamimoto, Andrei Iagaru, Mehran Jamali, Dawn Holley, Amir Barkhodari, Shreyas Vasanawala, Greg Zaharchuk

**Affiliations:** 1 Department of Radiology, Division of Nuclear Medicine and Molecular Imaging, Stanford University, Stanford, California, United States of America; 2 Department of Radiology, Molecular Imaging Program at Stanford, Stanford University, Stanford, California, United States of America; 3 Department of Radiology, Stanford University, Stanford, California, United States of America; Northwestern University Feinberg School of Medicine, UNITED STATES

## Abstract

**Purpose:**

To compare the conspicuity of malignant lesions between FDG PET/CT and a new simultaneous, time-of-flight (TOF) enabled PET/MRI scanner.

**Methods:**

All patients underwent a single-injection of FDG, followed by a dual imaging protocol consisting of PET/CT followed by TOF PET/MRI. PET/CT and PET/MRI images were evaluated by two readers independently for areas of FDG uptake compatible with malignancy, and then categorized into 5 groups (1: PET/MRI and PET/CT positive; 2: PET/MRI positive, PET/CT positive in retrospect; 3: PET/CT positive, PET/MRI positive in retrospect; 4: PET/MRI positive, PET/CT negative; 5: PET/MRI negative, PET/CT positive) by consensus. Patients with no lesions on either study or greater than 10 lesions based on either modality were excluded from the study.

**Results:**

Fifty-two patients (mean±SD age: 58±14 years) underwent the dual imaging protocol; of these, 29 patients with a total of 93 FDG-avid lesions met the inclusion criteria. The majority of lesions (56%) were recorded prospectively in the same location on PET/CT and PET/MRI. About an equal small fraction of lesions were seen on PET/CT but only retrospectively on PET/MRI (9%) and vice versa (12%). More lesions were identified only on PET/MRI but not on PET/CT, even in retrospect (96% vs. 81%, respectively; p = 0.003). Discrepant lesions had lower maximum standardized uptake value (SUV_max_) than concordant lesions on both modalities (p<0.001).

**Conclusions:**

While most lesions were identified prospectively on both modalities, significantly more lesions were identified with PET/MRI than with PET/CT.

## Introduction

Hybrid positron emission tomography / magnetic resonance imaging (PET/MRI) is one of the latest advances in multimodality technologies, and provides both biological and morphological information of malignant lesions [1]. Compared to PET/CT, the general advantages of PET/MRI are reduction of radiation exposure, use of MRI to image organ function, and improvement of diagnostic ability due to the better contrast of MRI imaging [2, 3]. Several studies have used combined data from separate PET and MRI examinations; however, these studies had limitations in terms of the time interval between studies and the potential for misregistration [2, 4]. Recent studies of simultaneous, non-time of flight (TOF) enabled PET/MRI scanners in clinical practice have shown promising initial results for several clinical indications [5–7].

Recently, a hybrid, whole-body 3T PET/MRI system with TOF PET capability has been developed [8]. By localizing counts to a shorter PET line-of-response (LOR), the TOF PET technique can reduce image noise, provide increased sensitivity and spatial resolution, and can mitigate potential errors caused by incorrect attenuation correction (AC) [9]. However, PET/MRI differs from PET/CT in terms of AC methods, image acquisition time, data processing, and image reconstruction. This study directly compares the sensitivity of PET/CT and TOF-enabled PET/MRI for detection of malignant lesions on ^18^F-fluorodeoxyglucose (FDG) examinations obtained for clinical purposes in oncology patients.

## Materials and Methods

### Patients

The Stanford Institutional Review Board approved this prospective study, and written informed consent was obtained from all patients. All patients were referred to the Division of Nuclear Medicine and Molecular Imaging for standard of care ^18^F-FDG PET/CT for initial or subsequent treatment strategy of malignancy. The inclusion criteria were 1) clinical indication for oncological PET/CT, 2) greater than 18 years of age, 3) ability to understand and hear instructions, 4) ability remain still for approximately 60 minutes duration of imaging, 5) ability to start PET/MR scan within 2 hrs from the end of the PET/CT scan, 6) < 55cm axial diameter and < 499 lbs. body weight. Exclusion criteria were 1) pregnancy, 2) metallic/conductive or electrically/magnetically active implants without MR safe or MR conditional labeling, 3) implants with MR unsafe labeling, 4) standard contraindications for MRI per screening policy of our hospital.

### PET/CT Scan Protocol

All patients fasted for at least 6 hours before injection of ^18^F-FDG, and blood glucose levels were less than 150 mg/dl at the time of the injection. All patients underwent a single-injection of ^18^F-FDG, with a dual-imaging protocol consisting of a PET/CT followed by PET/MRI. A standard diagnostic PET/CT examination was performed on a Discovery 600 PET/CT or Discovery 690 PET/CT scanner (GE Healthcare, WI, USA). For attenuation correction, low-dose helical CT (140 keV, 40 mAs, 512x512 matrix size) in shallow inspiration was performed of the same region as imaged by PET. The PET acquisition was performed in 3D mode with 3 minutes/bed position (47 slices/bed) in 6 beds with 11-slice overlap at the edge of the axial field-of-view (FOV). The PET images were reconstructed with a standard iterative algorithm (ordered subset expectation maximization [OSEM], 2 iterative steps and 32 subsets for Discovery 600 and 2 iterative steps and 24 subsets for Discovery 690).

### PET/MRI Scan Protocol

After the PET/CT scan, each patient was transferred to the whole-body, simultaneous, TOF- enabled 3.0 T PET/MRI (Signa PET/MR, GE Healthcare, Waukesha, WI, USA). Anatomic coverage was from the vertex to at least the mid-thighs, consistent with the PET/CT. The PET acquisition was performed in 3D mode with 4 min/bed position (89 slices/bed) in 5–9 beds with 15-slice overlap at the edges of the axial FOV. A 2-point Dixon 3-dimensional volumetric interpolated T1-weighted fast spoiled gradient echo image MR sequence (TR/TE1/TE2: 4.1/1.1/2.2 ms; FOV 50 x 37.5 cm; matrix 256 x 128; slice thickness/overlap: 5.2/2.6 mm; 120 images/slab; imaging time 18 sec) reconstructed in the axial plane was acquired at each table position and used to generate attenuation correction (AC) maps and for anatomic registration of the PET results. PET images were reconstructed using OSEM with 2 iterations and 28 subsets. The Dixon MRI sequence and the PET acquisition started at the same table position and times, thus ensuring optimal temporal and regional correspondence between MRI and PET data. For AC, the images were segmented into different tissue types differently in separate regions, and were co-registered to an atlas in the head region [9].

Additional sequences were acquired in the coronal plane as follows: short tau inversion recovery (STIR) images (TR/TI/TE: 4300/ 190/44.2 ms; FOV 44–46 cm; matrix 384 x 224; slice thickness/skip: 8/0 mm; 22–38 slices depending on size; 2 nex; acceleration factor 2; imaging time 1:52–7:11 min) and liver acquisition with volume acquisition (LAVA) images (3D spoiled gradient echo; TR/TE1/TE2: 4.9/1.3/2.5 ms; FOV 44 cm; matrix 320 x 224; slice thickness/ overlap: 4/2 mm; 88–152 slices depending on size; 2 nex; acceleration factor 2; imaging time 0:21–0:55 min), which allowed for water and fat separation, were acquired in each alternating bed position (e.g., 1, 3, 5, etc.). Because of the large FOV, this allowed coverage of both the current and subsequent bed position, such that a full body image could be created from these sequences. In the thorax region, the MRI scans were acquired during breath-hold in shallow inspiration.

### Image Analysis

Two board certified readers (a nuclear medicine physician and radiologist, respectively) reviewed PET/CT and PET/MRI images for FDG uptake considered consistent with malignancy in a separate and independent fashion. To more closely simulate clinical proactive, the patient’s clinical history and previous PET/CT images (if available) were used to help guide the interpretation. The readers were otherwise blinded to any additional information.

For rating the PET/CT data, PET images were screened for any focal uptake suggesting malignancy, and the PET fused low-dose CT scan was used for anatomic correlation. PET/CT review was performed on non-TOF reconstructed images, which is the usual practice at our medical center. The center of the identified FDG uptake was recorded by location (x, y, and z) for subsequent comparison. For rating the PET/MRI studies, PET images were screened for any focal uptake suggesting malignancy, and all available MR imaging sequences were used for anatomic correlation, as needed. Similar to the PET/CT method, the center of the identified FDG uptake was recorded by location (x, y, and z) for subsequent comparison. In patients with multiple lesions, we excluded cases with over 10 lesions per patients identified either by PET/MRI or PET/CT in order to avoid biasing the results by relatively few subjects with many lesions. Also, cases with many FDG avid lesions are found in patients with advanced metastatic cancer, where the precise number of identified lesions would not significantly impact management.

After this separate review was complete, a consensus session was held to compare the results from the PET/CT and PET/MRI studies. These were reviewed together by the two readers and categorized into 5 groups (1 = both PET/MRI and PET/CT positive prospectively; 2 = PET/MRI positive prospectively, PET/CT positive in retrospect; 3 = PET/CT positive prospectively, PET/MRI positive in retrospect; 4 = PET/MRI positive prospectively, PET/CT negative [even at the consensus session]; 5 = PET/MRI negative [even at the consensus session], PET/CT positive prospectively). Then, we measured the maximum standardized uptake value (SUV_max_) of the identified FDG uptake by placing volumes of interest (VOIs) over the lesions.

### Statistical Analysis

Wilcoxon signed rank test was used to evaluate the differences of FDG uptake (SUV_max_) between PET/CT and PET/MRI for the suspicious FDG lesions. The Mann–Whitney U test was used to evaluate the difference of FDG uptake (SUV_max_) between concordant and discordant lesions in PET/CT or PET/MRI. Differences in lesion detection between PET/CT and PET/MRI were compared by the McNemar test. Statistical analyses were performed using the statistical package Stata (version IC 11; Stata Corp., TX, USA). A p-value of p<0.05 was considered statistically significant.

## Results

**Fig 1:**
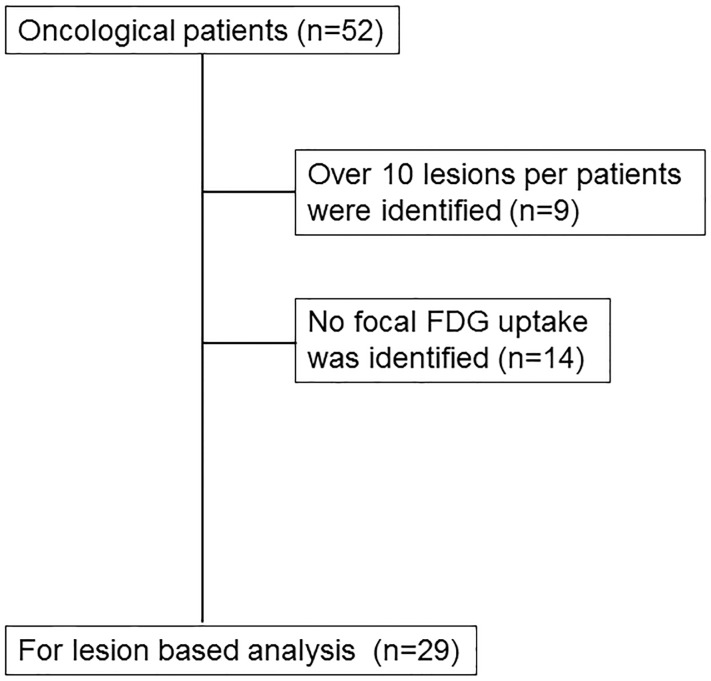
Flow chart illustrating the inclusion of cases in this study.

Between January 2014 and February 2015, 52 patients were recruited consecutively in this prospective study (mean age, 58±14 years [range 27–86 years]; 25 male, 27 female). 11.5% of the participants were referred for initial treatment strategy, the rest were referred for subsequent treatment strategy (treatment monitoring, restaging and detection of suspected recurrence, etc.) [10]. More than 10 lesions were identified either by PET/MRI or PET/CT in 9 patients; these patients were excluded from analysis. In 14 patients, no focal FDG uptake suggesting malignancy was detected on either PET/CT or PET/MRI (Fig 1).

There were no cases in which lesions were seen on one modality but not the other. Therefore, subsequent lesion-based analyses were based on the remaining 29 patients. Further characteristics of the patient population are found in Table 1.

**Table 1:** Clinical characteristics of the patient population.

Index	Number	Ratio (%)
Number		
Total	29	
Male	14	48.3
Female	15	51.7
Age [mean ± SD, (range)]		
Total	56 ± 14 (27–79 yrs)	
Male	57 ± 11 (32–72 yrs)	
Female	54 ± 17 (27–79 yrs)	
Reason for PET/CT study		
Diagnosis and initial staging	4	13.8
Subsequent treatment strategy	25	86.2
Primary lesion		
Lymphoma	14	48.3
Head and neck cancer	3	10.3
Breast cancer	2	6.9
Lung cancer	2	6.9
Colorectal cancer	2	6.9
Neuroendocrine tumor	2	6.9
Melanoma	1	3.4
Mesothelioma	1	3.4
Pancreas cancer	1	3.4
Bladder cancer	1	3.4

PET/CT started 68 ± 15 min (range: 46–104 min) after injection of 10.0 ± 1.0 (range: 8.1–12.0) mCi of FDG, while PET/MRI started 148 ± 23 min (range: 100–184 min) after injection of FDG, with the mean time between examinations being 59 ± 16 min (range: 28–100 min). The average length of the PET/CT scan was 22 ± 7 min (range: 10–37 min), while the average length of the PET/MRI scan was 62 ± 15 min (range: 35–88 min) (p<0.001).

**Fig 2:**
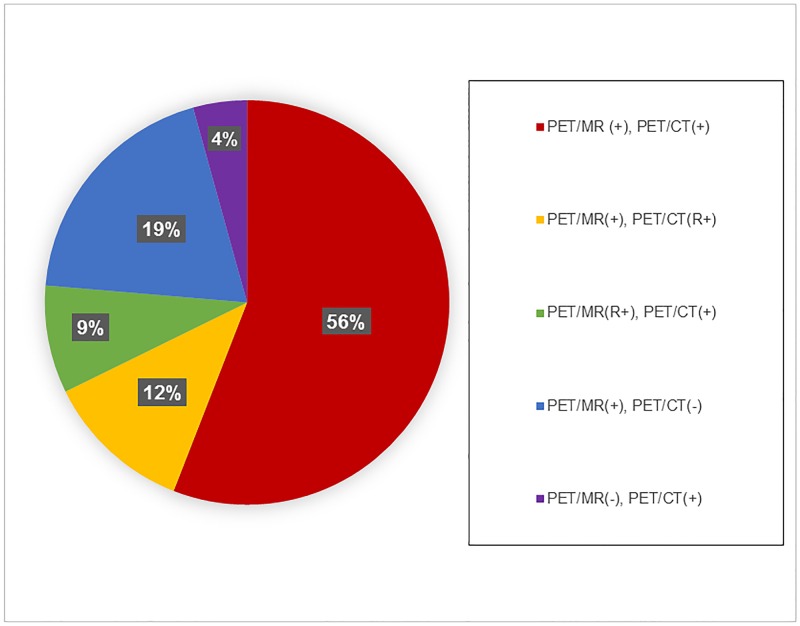
Classification of PET/MRI and PET/CT interpretations for presumed malignant lesions.

Fig 2 summarizes the data regarding presumed malignant lesions. In total, 93 lesions were identified either on PET/MRI or PET/CT. In the majority of cases (55.9%), the same lesion was detected prospectively using each modality. In most of the discrepant cases, the lesion was visible in retrospect on the other modality (20.4% of lesions overall, 46.3% of discrepant lesions). The smallest categories were those in which a lesion was seen on one modality but not visible even in retrospect on the other modality (18.4% of lesions on PET/CT, and 4.3% of lesions on PET/MRI). As a result, PET/MRI detected 95.7% of all lesions, which was significantly higher than PET/CT, which detected 81.6% of all lesions (p = 0.003).

PET/MRI and PET/CT findings by anatomical location are shown in Table 2.

**Table 2:** Result by the location and lesion type.

Subject	PET/MRI (+) PET/CT (+) (n = 52)	PET/MRI (+) PET/CT (R+) (n = 11)	PET/MRI (R+) PET/CT (+) (n = 8)	PET/MRI (+) PET/CT (-) (n = 18)	PET/MRI (-) PET/CT (+) (n = 4)	Total (n = 93)	
						PET/MRI (+)	PET/CT (+)
Anatomical Location						89	75
Brain	0	0	0	1	0	1	0
Head and neck	8	0	1	6	0	15	9
Chest	20	1	2	2	1	25	24
Abdomen	8	4	4	5	2	21	18
Pelvis	16	6	1	4	1	27	24
Lesion type						89	75
Extra nodal lesion	11	0	0	6	2	17	13
Nodal lesion	38	10	8	12	2	68	58
Skeletal lesion	3	1	0	0	0	4	4

R+: positive on retrospective review

PET/MRI identified more suspicious FDG lesions than PET/CT in all anatomical locations. The suspicious FDG lesions observed prospectively in the same location for both PET/CT and PET/MRI were highest in the chest area and lowest in the abdomen. SUV_max_ values for different lesion types are shown in Table 3.

**Table 3:** Result of quantitative value (SUVmax) from PET/MRI and PET/CT.

Subject	PET/MRI (+) PET/CT (+) (n = 52)	PET/MRI (+) PET/CT (R+) (n = 11)	PET/MRI (R+) PETCT (+) (n = 8)	PET/MRI (+) PET/CT (-) (n = 18)	PET/MRI (-) PET/CT (+) (n = 4)	Total (n = 93)	
						Concordance (n = 52)	Discrepant (n = 41)
PET/MRI	8.7 ± 5.8	4.0 ± 2.3	4.7 ± 1.7	3.6 ± 2.6	-	8.7 ± 5.8 *	4.0 ± 2.3
	(2.6–30.8)	(1.9–8.2)	(2.3–6.9)	(1.3–12.7)		(2.6–30.8)	(1.9–12.7)
PET/CT	6.6 ± 5.0	2.4 ± 0.7	4.3 ± 1.9	-	3.9 ± 0.6	6.6 ± 5.0 *	3.2 ± 1.5
	(2.3–27.0)	(1.4–3.5)	(1.7–6.7)		(3.7–4.3)	(2.3–27.0)	(1.4–6.7)

R+: positive on retrospective review. Range of SUVmax was shown in parenthesis.

*Concordant lesions had significantly higher SUVmax values than discrepant lesions (p<0.001)

**Fig 3:**
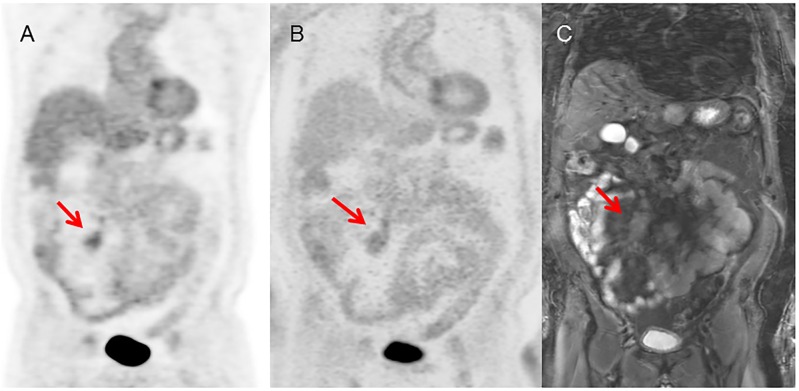
Concordant lesion, identified prospectively on both modalities. 66 year-old male with neuroendocrine tumor who underwent PET/CT for initial staging. (A) PET image from PET/CT, (B) PET image from PET/MRI, (C) MRI (STIR) image. Both PET/CT and PET/MRI could identify the mesenteric lesion (arrow).

**Fig 4:**
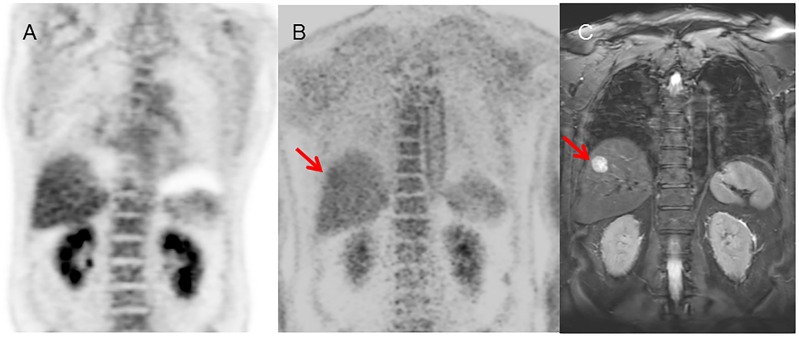
Lesion identified prospectively on PET/MRI but only retrospectively on PET/CT. 66 year-old male with neuroendocrine tumor who underwent PET/CT for initial staging (same patient as in Fig 3). (A) PET image from PET/CT, (B) PET image from PET/MRI, (C) MRI (STIR) image. This liver metastasis could be clearly identified on PET/MRI because of the T2-bright lesion seen on MR STIR imaging. The lesion has only mildly increased activity compared to the normal liver, and was not evident prospectively due to the poor soft tissue contrast of the CT scan from the PET/CT study.

**Fig 5:**
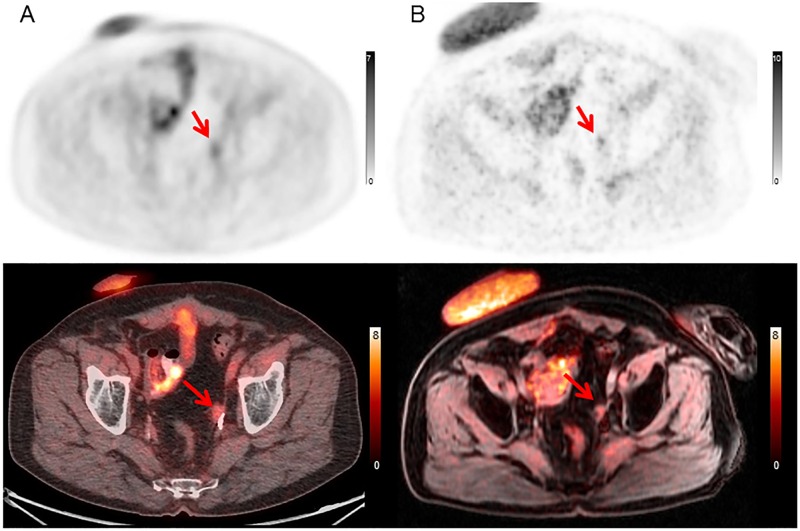
Lesion identified prospectively on PET/CT but only retrospectively on PET/MRI. 65 year-old male with bladder cancer who underwent PET/CT for subsequent treatment strategy. (A) PET/CT image (upper: PET image from PET/CT, lower: fused PET and CT) (B) PET/MRI image (upper: PET image from PET/MRI, lower: fused PET and MRI). PET/CT could identify the small nodular lesion at the left pelvic wall, due primarily to the better spatial localization possible with CT in this case. It was identified on PET/MRI only retrospectively, as on the initial read, it was thought to be activity in adjacent bowel.

**Fig 6:**
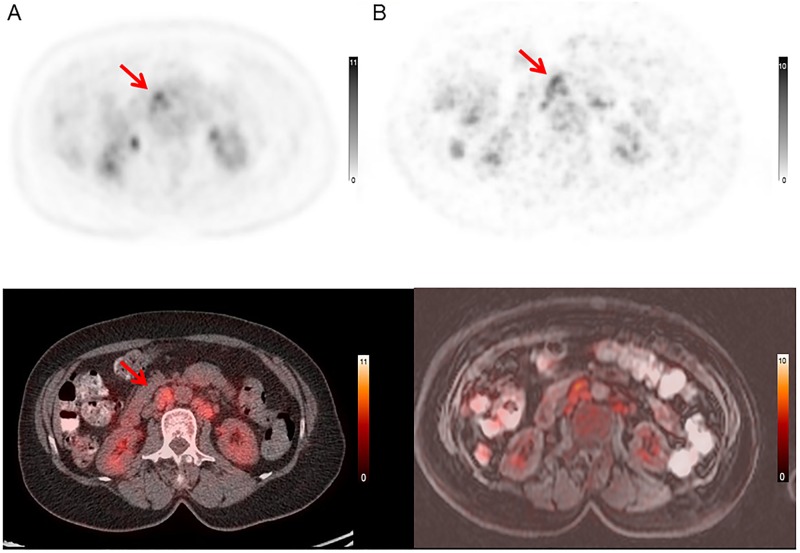
PET/MRI negative, PET/CT positive case. 69 year-old female with malignant lymphoma who underwent PET/CT for initial staging. (A) PET/CT image (upper: PET image from PET/CT, lower: fused PET and CT) (B) PET/MRI image (upper: PET image from PET/MRI, lower: fused PET and MRI). PET/CT could identify the para-aortic lymph nodes. In PET/MRI, PET/MRI could not distinguish these lesions from physiological FDG uptake in the small intestine, even in retrospect.

**Fig 7:**
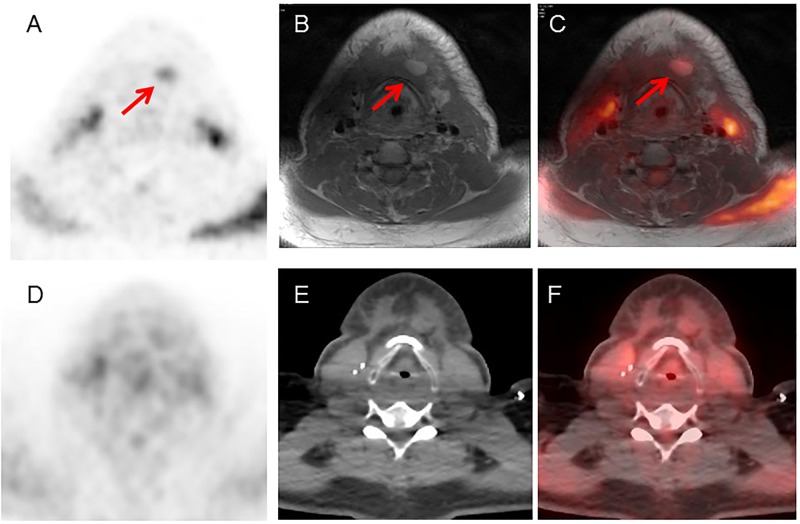
PET/MRI positive, PET/CT negative case. 32 year-old male with tongue cancer who underwent PET/CT for subsequent treatment strategy. (A) PET image from PET/MRI, (B) MRI image (T1-weighted image), (C) PET and MRI fused image, (D) PET image from PET/CT, (E) CT image, (F) PET and CT fused image. PET/MRI could detect the submandibular lymph node clearly, both because of better lesion to background contrast, but also due to superior MR contrast. PET/CT showed no abnormal uptake in this lesion, even though the node itself could be seen on PET/CT.

PET in PET/MRI demonstrated higher SUV_max_ than PET in PET/CT in concordant lesions (p<0.001), consistent with previous report [8]. Discrepant lesions had lower SUV_max_ values than concordant lesions for both modalities (p<0.001). The discrepant lesions with the highest SUV_max_ included three para-aortic lymph nodes (SUV_max_ = 6.4–6.7) that were not prospectively detected by PET/MRI (SUV_max_ = 6.1–6.9), as they were indistinguishable from physiological FDG uptake in the small intestine. Two FDG-avid mass lesions in the abdominal wall were not prospectively detected by PET/CT (SUV_max_; PET/MRI: 5.3–6.0, PET/CT: 1.4–3.3), again due to the difficulty of delineating the abnormal uptake from physiological FDG uptake in the ascending colon. One pelvic lymph node was not prospectively detected by PET/CT (SUV_max_; PET/MRI: 8.2, PET/CT: 3.5), and one brain lesion (SUV_max_ = 12.7 in PET/MRI) was not identified by PET/CT. Other than these lesions, there were no discrepant lesions when SUV_max_ was >5 on either modality. Representative cases for each of the categories are shown in Figs 3–7.

## Discussion

This study directly compares the sensitivity of PET/CT and TOF-enabled PET/MRI for detection of malignant lesions on FDG examinations obtained for clinical purposes in oncology patients. TOF PET/MRI provided comparable diagnostic ability with PET/CT, despite decreased FDG activity for imaging at a later time point. Significantly more lesions were identified with PET/MRI than with PET/CT. The reasons for this are not entirely clear, but may relate to improved lesion-to-background at later imaging times, the increased sensitivity of the TOF PET detectors in PET/MRI, longer imaging times that are possible due to the need to acquire MR information, and the superior soft tissue contrast afforded by the simultaneous MR imaging. Given the structure of the study, it is difficult to disentangle these factors and, in any case, it is likely to be multifactorial. Perhaps it is not surprising that PET/MRI performed better than PET/CT given that the PET/CT was performed without a diagnostic CT, while the PET/MRI, in contrast, included multiple diagnostic MR sequences. However, each method of review is consistent with the most commonly used approach for that modality at the current time, so we believe this simulates the clinical situation accurately.

In a previous study using a TOF-enabled simultaneous scanner, PET/MRI also showed comparable performance with PET/CT, despite the fact that images were acquired over 2 hours after FDG injection. This study also suggested that PET/MRI could detect all the lesions identified on the PET/CT, though the methodology for image interpretation was different from that of the current study [8].

In our study, the suspicious lesions in the chest area were highly concordant on both modalities. Because our patient population had a high proportion of lymphoma patients, the vast majority of these were lymph nodes in the hila, which were more conspicuous on PET/MRI; only a few lung nodules were identified on either modality in our cohort, and it remains an open question of whether PET/MRI is sufficient to properly stage patients with lung metastases. In contrast, it was more common that discrepant lesions were found in the head and neck region, which may be due to the higher sensitivity of PET/MRI for small neck lymph nodes, which was aided greatly by better soft tissue MR contrast, especially the use of diffusion and T2 fat-saturated imaging.

A major advantage of MRI compared to CT is the ability to provide better soft-tissue contrast. Another potential advantage is the ability to acquire physiological information with functional MRI, MR spectroscopy, diffusion imaging, and perfusion imaging. Therefore, PET/MRI may have great potential for improving diagnostic ability by combining it with this additional information obtained from MRI. For example, we found that PET/MRI (but not PET/CT) could detect a liver metastasis with FDG uptake only slightly higher than that of normal liver (Fig 4), consistent with prior reports of higher PET/MRI sensitivity than PET/CT for detecting liver lesions [11, 12]. We did not apply any advanced functional imaging beyond diffusion, but other studies do suggest that further enhancement of detection and characterization of lesions is possible with modalities, such as dynamic contrast enhancement. However, one limitation of PET/MRI that has been raised with dynamic contrast-enhanced MRI is that the lower specificity of PET/MRI may lead to incorrect management decisions [9].

As expected, discordant lesions tended to have lower SUV_max_ values. Therefore, one explanation for PET/MRI’s better performance may have been the added sensitivity of the TOF PET scanner, which was measured to be about 2-fold higher than the PET/CT scanners used in this study [13]. For cases seen on PET/CT but not on PET/MRI even in retrospect, the primary commonality was the difficulty in localizing the PET/MR activity on the lower spatial resolution MR images, and in particular to distinguish lesions from activity within the small and large bowel. It is hoped that dedicated MR sequences, including variable flip angle refocused single-shot fast spin echo [14] and compressed sensing approaches will lead to improved visualization of this important distinction within the abdomen and pelvis to further improve PET/MRI performance. Given that TOF PET performance can reduce image noise and improve spatial resolution, the issue of precise localization of abnormal activity will become crucial [9]. The pilot study showed that SUV measurement was higher in PET/MRI than those in PET/CT [8], possibly due to redistribution and influenced by the use of MR attenuation correction. Despite the loss of signal from radiotracer decay, comparison of the PET images from PET/CT and PET/MRI show no loss of image quality, probably related to increased signal-to-background as well as the superior detector quality of the TOF PET/MRI.

The length of PET/MRI scan was longer than that of PET/CT due to the addition of multiple diagnostic MR sequences. Although PET/MRI did identify more lesions than PET/CT, the patient was required to keep still in PET/MRI scanner longer than in PET/CT, which may not be possible for all patients. It is difficult to estimate how long it took for the interpretation of PET/MRI compared to PET/CT. The time will depend on scan range, the number of MRI sequences, the ability and experience of the reader. Moreover, it is still challenging to determine standardized approaches in interpretation to avoid missed lesions in both modalities.

There are several limitations to this study. These include the inability to randomize the order of the PET/CT and PET/MRI, such that we could not determine the role of tracer redistribution and decay on image quality. This was due to the regulatory constraints of the Investigational New Device (IND) protocol that was used to acquire the imaging cases, which required that PET/CT be performed at its standard time. We report a relatively small number of cases, though we did see a significant number of lesions, and the findings were statistically significant; we doubt that a larger study would come to a significantly different conclusion. Again, because of the constraints of the IND, we could not collect additional cases. While the cases were acquired in a prospective fashion, the case mix was skewed towards lymphoma, due to the referral patterns at our institution. For that reason, performance in identifying activity in lymph nodes played a large role in our findings. However, for both nodal and extra-nodal lesions, the same pattern of improved visualization with PET/MRI was seen.

TOF PET/MRI provided comparable diagnostic ability with PET/CT, using typical clinical imaging protocols, despite imaging at a later time point. Significantly more lesions were independently identified with TOF PET/MRI than with PET/CT. In general, discordant lesions had lower SUV_max_, but discrepancies were seen in some higher activity lesions on each modality due to challenges in separating malignant lesions from physiological small and large bowel activity, suggesting improved abdominal MR imaging may yield benefits for future studies. Limitations of this study include the relatively small number of patients and the differences in the PET imaging timelines. The time duration between PET/CT and PET/MRI was caused because the different scanners were located in separate buildings at our facility. Further work may be helpful for evaluating specific oncological indications in more detail and to determine which factors are responsible for this improved performance, in particular the role of TOF PET/MRI imaging.

## Conclusion

While most lesions were identified prospectively on both modalities, significantly more lesions were identified with PET/MRI than with PET/CT.
